# Associations of chemo- and radio-resistant phenotypes with the gap junction, adhesion and extracellular matrix in a three-dimensional culture model of soft sarcoma

**DOI:** 10.1186/s13046-015-0175-0

**Published:** 2015-06-10

**Authors:** Chujie Bai, Min Yang, Zhengfu Fan, Shu Li, Tian Gao, Zhiwei Fang

**Affiliations:** Department Bone and Soft Tissue Tumor, Key Laboratory of Carcinogenesis and Translational Research (Ministry of Education), Peking University Cancer Hospital and Institute, Beijing, 100142 People’s Republic of China; Department of Gerontology, Beijing Shijitan Hospital, Capital Medical University, Beijing, 100038 People’s Republic of China

**Keywords:** Soft sarcoma, Three-dimensional culture, Primary culture, Human osteosarcoma cell line 1 (HOSS1), Chemo-resistance, Radio-resistance

## Abstract

**Background:**

Three-dimensional (3D) culture models are considered to recapitulate the cell microenvironment in solid tumors, including the extracellular matrix (ECM), cell-cell interactions, and signal transduction. These functions are highly correlated with cellular behaviors and contribute to resistances against chemo- and radio-therapies. However, the biochemical effects and mechanisms remain unknown in soft sarcoma. Therefore, we developed an in vitro 3D model of sarcoma to analyze the reasons of the chemo- and radio-resistance in therapies.

**Methods:**

Four soft sarcoma cell lines, HT1080, RD, SW872, and human osteosarcoma cell line 1 (HOSS1), a cell line established from a patient-derived xenograft, were applied to 3D culture and treated with growth factors in methylcellulose-containing medium. Spheroids were examined morphologically and by western blotting, RT-qPCR, and immunofluorescence staining to analyze cell adhesion, gap junctions, ECM genes, and related factors. Proliferation and colony formation assays were performed to assess chemo- and radio-resistances between 3D and two-dimensional (2D) cell cultures. Annexin V and Propidium Iodide staining was used to detect early apoptotic sarcoma cells treated with Doxorubicin, Gemcitabine, and Docetaxel in the 3D model.

**Results:**

The four soft sarcoma cell lines formed spheres in vitro by culture in modified condition medium. Compared with 2D cell culture, expression of ECM genes and proteins, including *COL1A1*, *LOX*, *SED1*, *FN1*, and *LAMA4*, was significantly increased in 3D culture. Analysis of cadherin and gap junction molecules showed significant changes in the gene and protein expression profiles under 3D conditions. These changes affected cell–cell communication and were mainly associated with biological processes such as cell proliferation and apoptosis related to chemo- and radio-resistances.

**Conclusions:**

Our findings revealed significant differences between 3D and 2D cell culture systems, and indicated that cellular responsiveness to external stress such as radiation and chemotherapeutics is influenced by differential expression of genes and proteins involved in regulation of the ECM, cell adhesion, and gap junction signaling.

**Electronic supplementary material:**

The online version of this article (doi:10.1186/s13046-015-0175-0) contains supplementary material, which is available to authorized users.

## Introduction

Soft tissue sarcomas are malignant tumors of mesenchymal origin, among which rhabdomyosarcoma [1], fibrosarcoma [2], and osteosarcoma [3], are the most common histological subtypes in childhood and adolescence. Despite advances in cancer therapy, the mainstay of treatment for this disease is still surgical resection. Despite conventional chemotherapy and radiation therapy, the overall survival of patients with soft sarcomas has not improved in the past 50 years [4, 5]. The prognosis of soft sarcomas remains one of the worst amongst solid tumors in children [6]; consequently, soft sarcoma has become an increasingly researched cancer over the past few decades.

Traditionally, cancer researchers rely on two-dimensional (2D) in vitro studies and small animal models to examine the complex mechanisms of cancer cell proliferation, apoptosis, angiogenesis, invasion, and metastasis. Typically, the arrangement of the cells in 2D primary culture permits less cell—cell interaction and absent ECM secretion, with concomitant effects on gene and protein expression and cellular behavior [7, 8]. Therefore, a novel method of culturing tumor cells, three-dimensional (3D) culture, was introduced in the 1970s [9]. This model allowed researches to focus on the cellular morphology and interactions between tumor cells. Cell–cell and cell-ECM interactions observed during in vivo tumor progression cannot be studied in 2D models, whereas 3D models are capable of mimicking these conditions. Subsequently, various sarcoma cell lines have been tested for their ability to grow as spheroids in 3D culture [10–12]. Many studies have verified that 3D cultures of epithelial tumor cell lines are generally more resistant to chemo- and radio-therapies than their 2D counterparts [13–15]. Chemo-resistance in tumors is mediated by various mechanisms. The classical mechanisms are based on ATP-binding cassette (ABC) transporter proteins, particularly ABCB1 (MDR1), ABCC1 (MRP1), and ABCG2 (BCRP1), all of which have been reported to contribute to chemo-resistance in soft sarcoma cells [16]. However, validated 3D in vitro sarcoma cell models have not been developed for rapid and standardized screening of chemo- and radio-resistances.

In the present study, we developed an in vitro alginate scaffold 3D model of sarcoma using HT1080 (fibrosarcoma), SW872 (liposarcoma), and RD (rhabdomyosarcoma) cell lines, as well as human osteosarcoma cell line 1 (HOSS1; osteosarcoma) that was primary cultured from a patient-derived xenograft (PDX). Anticancer effects of various chemotherapeutic agents were studied and IC_50_ values were compared between conventional 2D and 3D cell cultures. We provide evidence of cell adhesion mediating chemo-resistance, a new hypothesis relating chemo-resistance to the microenvironment such as the stroma and ECM, which was proposed by Hazlehurst et al. [17]. In addition, we found that sarcoma cells in the 3D model were more radio-resistant, and observed more stromal rich or adhesion-rich phenotypes. A better understanding of how these tumors originate and progress is needed for the development of targeted sarcoma therapies.

## Methods

### Cell culture

The following well-characterized human sarcoma cell lines (American Type Culture Collection) were used in this study: RD, HT1080, and SW872. We also used HOSS1 cells as described in Additional file 1: Supplemental Method. The cells were cultured under standard culture conditions (5 % CO_2_ at 37 °C) in Dulbecco’s modified Eagle’s medium (DMEM)/F12 or phenol red-free DMEM/F12 (Gibco, USA) containing 10 % fetal bovine serum (FBS, Invitrogen, USA).

### 3D culture

Cells were treated with trypsin and counted. Subsequently, the cells were seeded into round-bottom Ultra-Low Attachment 96 well-plates (Corning, Acton, USA) at 200 cells/well in 100 μl DMEM-F12 or phenol red-free DMEM-F12 containing 20 ng/ml epidermal growth factor (EGF), 20 ng/ml basic fibroblast growth factor (bFGF), 10 ng/ml hepatocyte growth factor (HGF), 10 % B27, 2 % bovine pituitary extract (BPE), and 20 % methylcellulose as described in Supplemental Method. Spheroids were grown under standard culture conditions (5 % CO at 37 °C) and harvested at various time points for RNA isolation or drug testing as described below.

### Cell proliferation assay

3D culture spheres or 2D culture clones were collected and trypsinized with 0.02 M EDTA to single cell. Cells were counted with Countess (Life Technologies, USA) each two days. Cell generation time (GT = Tn/5; Tn = t/ ln (Nf/Ni)/ ln2; Nf: final cell number at time t; Ni: initial plated cell number; ln: natural logarithm, 5 replicates) was calculated by plotting the graph of cell counting versus time. This experiment was repeated in triplicate.

### cDNA microarray

Equal amounts of cDNA from HOSS1 cultured in 2D and 3D cells were labeled with Cy3, respectively, and were then carried out hybridization according to the manufacture’s protocol (Affymetrix) using Affymetriix human genome gene chip HG133 Plus 2.0 (Oebiotech Co, Ltd., Shanghai, China). Genes were considered to be up or down-regulated when the fluorescent intensity ratio between 2D and 3D cells was greater than 2 or less than 0.5. The experiment was repeated once. Hierarchical clustering of regulated genes in this study was measured by Genespring software.

### RNA isolation and RT-qPCR analysis

Cells or spheroids were collected, washed once with cold PBS, and processed for total RNA isolation using an RNeasy Mini Kit (Qiagen, Germany). RNA integrity and concentrations were analyzed by agarose gel electrophoresis and a Nano-drop Spectrophotometer. One microgram of total RNA was reverse transcribed by M-MLV (Invitrogen, USA) (see Supplemental Method). SYBR-Green Technology (Toyobo, Japan) was used for all RT-qPCR experiments. Further detailed information regarding RT-qPCR is provided in Supplemental Method. Primers were designed by the PCR primer database of Gene Runner and purchased from Invitrogen, which is described in Additional file 2: Table S1.

### SDS-polyacrylamide gel electrophoresis (PAGE) and western blotting

Cell lysates of 2D and 3D cultures were prepared using RIPA lysis buffer. The protein concentrations were measured using a BCA Protein Assay kit (Pierce, USA). Proteins samples (50 μg) were resolved by 10 % SDS-PAGE and electrophoretically transferred to PVDF membranes. The membranes were blocked with 5 % fat-free dry milk in TPBS and then incubated for 1 h at room temperature with primary antibodies (see Additional file 2: Table S2). The primary antibodies were detected with peroxidase-conjugated goat anti-rabbit or anti-mouse IgGs (Jackson, USA). The signals were detected by enhanced chemiluminescence (Pierce).

### Immunofluorescence staining

Spheroids were harvested at various time points and washed twice with PBS. For immunohistochemistry, spheroids were fixed in 4 % paraformaldehyde, embedded in paraffin, and sectioned at 7 μm thicknesses. Prior to blocking in PBS plus Tween and 1 % bovine serum albumin, the sections were boiled in 0.01 M sodium citrate buffer (pH 6.0) for antigen retrieval. Anti-E-cadherin and anti-N-cadherin antibodies were used as primary antibodies. FITC-labeled anti-rabbit or anti-mouse secondary antibodies (Jackson) were used to detect primary antibodies using a Leica SP5 laser scanning confocal microscope.

### Drug treatments

For 2D culture, cells were seeded in flat-bottom 48-well plates (Costar, USA) at 2500 cells/well in 100 μl RPMI-1640 medium containing 10 % FBS. For 3D culture, cells were seeded according to the description for spheroid preparation in complete medium. Doxorubicin (AstraZeneca, UK), Gemcitabine (Lilly, France), and Docetaxel (Sanofi, France) were added to the medium of eight replicates for each time point at the indicated final concentrations.

### Radiation resistance assay

For 2D culture, a colony formation assay was performed to determine the sensitivity of cells to X-ray irradiation. Two hundred cells were seeded in each 35-mm dish and allowed to attach. The cells were then exposed to 0–8 Gy X-ray radiation. The control group did not receive any exposure. Colonies were fixed in methanol and stained with Giemsa at day 14 after exposure. A minimum of 50 viable cells were scored as a colony. For 3D culture, cells were seeded in Ultra-Low Attachment 96-well plates according to the above description. Spheroid formation was analyzed at 2 weeks after exposure to 0–8 Gy X-ray radiation. Cell survival fraction (SF) was based on the formula: Ne/ (Ce × N0/ C0 × 100 %), Ne: Colony numbers in experimental group; Ce: Plating numbers in experimental group; N0: Colony numbers in control group; C0: Plating numbers in control group. Cell survival curve was obtained by the single-hit multi-target model (SF = l-(1-e-Do/D) N) using GraphPad Prism 5 version. The parameters of cell survival fraction mean lethal dose value (Do) and extrapolation number (N) were calculated with a 2 Gy irradiation dose (SF2). Higher values indicated a higher radio-resistance.

### Measurement of apoptosis

Cells were stained with Annexin V-FITC and Propidium iodide (PI) according to the manufacturer’s instructions (Roche, Germany) to determine the percentage of cells undergoing apoptosis. Annexin V and PI staining was assessed by Flow Cytometry (Becton Dickinson, CA, USA). The percentage of apoptotic cells was expressed as the percentage of the total cell population. To confirm the reliability of this assay for 3D-cultured cells, spheroids were disaggregated with TrypLE Express (Invitrogen) without chelators for 15 min at 37 °C, re-suspended as single cells, and analyzed by Flow Cytometry.

### Statistical analysis

All data were analyzed by SPSS 11.0 statistical software for Windows. The statistical differences in the data were analyzed using a Student’s *t*-test with two sided of at least 5 replicates. All experiments were performed at least three times. A *p*-value of less than 0.05 was regarded as statistically significant.

## Results

### Formation of compact 3D spheroids by soft sarcoma cell lines

**Fig. 1:**
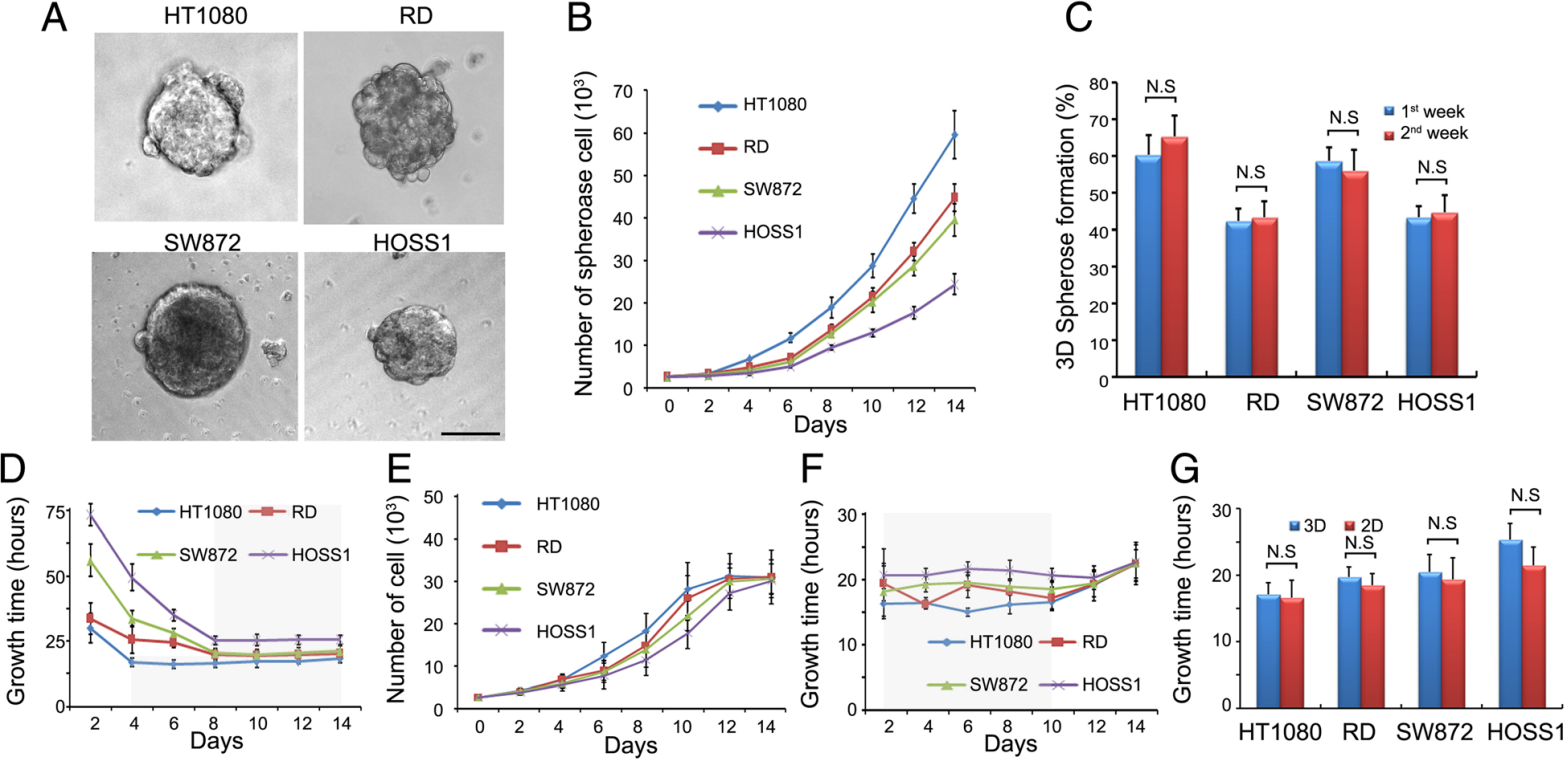
Effects of spheroid formation and growth. **a**: HT1080, RD, SW872 and HOSS1 soft sarcoma cell lines grown in 3D culture at day 8. HOSS1 cells established from an osteosarcoma PDX were also grown in special medium without FBS. **b**: Cell counts in 3D cultures at various time points. **c**: Cell counts in 3D spheres between weeks 1 and 2 were not significantly different (range: 40–65 %). **d** Stable proliferation of the four cell lines in 3D culture. HT1080 cells required 4 days, whereas the other cell lines needed 8 days. The four cell lines underwent stable growth after these initial periods. **e**, **f**: Cell numbers and growth time in 2D cultures at various times. In contrast to 3D cultures, cells did not lose stability until 10 days in 2D culture. Shaded areas indicate stable proliferation. **g**: Doubling times of 2D and 3D cell cultures at day 8 after plating. Results are the mean ± SD (*n* = 6). N.S., not significant, Bar = 100 μm

After successfully cultured HOSS1 cell line (see Additional file 3: Figure S1), followed by supplements for 3D culture [18], we grew the four sarcoma cells in medium containing methylcellulose, a cellulose-derived inert compound that enhances spheroid formation, instead of exogenous ECM components (Fig. 1a). We evaluated HT1080, RD, SW872, and HOSS1 cell lines for their ability to form spheroids (Fig. 1b, c). At 8 days after plating, all cell lines were undergoing stable exponential growth with similar doubling times (Fig. 1d). This similarity in doubling times provided comparable experimental conditions for gene and protein expression analyses. In 3D culture, HT1080 cells reached stable proliferation within 4 days, but RD, SW872 and HOSS1 cells required 8 days. All cell lines reached stable proliferation within 48 h in 2D culture (Fig. 1e, f). The growth kinetics of spheroid formation was assessed longitudinally. Cells were counted in 2D and 3D cultures at various time points. Compared with conventional 2D culture, the increase in cell numbers over time indicated no reduction in the proliferation of cells in 3D culture (Fig. 1g).

### Identification of genes regulated in 2D and 3D cultures

To gain further insight into the possible mechanisms underlying 3D culture compared with 2D, we performed cDNA microarray analyses to find global gene expression changes in 3D system versus 2D. From our analysis, 116 genes were classified as being up-regulated genes with a ratio ≥2.0, and 94 genes were classified as being down-regulated with a ratio ≤0.5 (see Additional file 2: Table S3). Those genes reportedly involved in tumor cell adhesion, gap junction and ECM, such as CHD1, Cx26, Cx43, Cx45, COL1A1, FN1, LOX, ITGB1 and LAMA4, were also up-regulated following 3D cultures. Interestingly, ABC transport genes such as ABCB1, ABCC1, ABCC3, ABCG2, functional chemo-resistance by pumping out drug molecules, were also enhanced in 3D cultures. We were next interested in expression of cadherin, gap junctions, ECM in 3D system according to sensitive ABC transport and anti-apoptosis genes reflected chemo-resistance in 3D platform.

### Expression of adhesion and gap junction molecules in 3D cultures

**Fig. 2:**
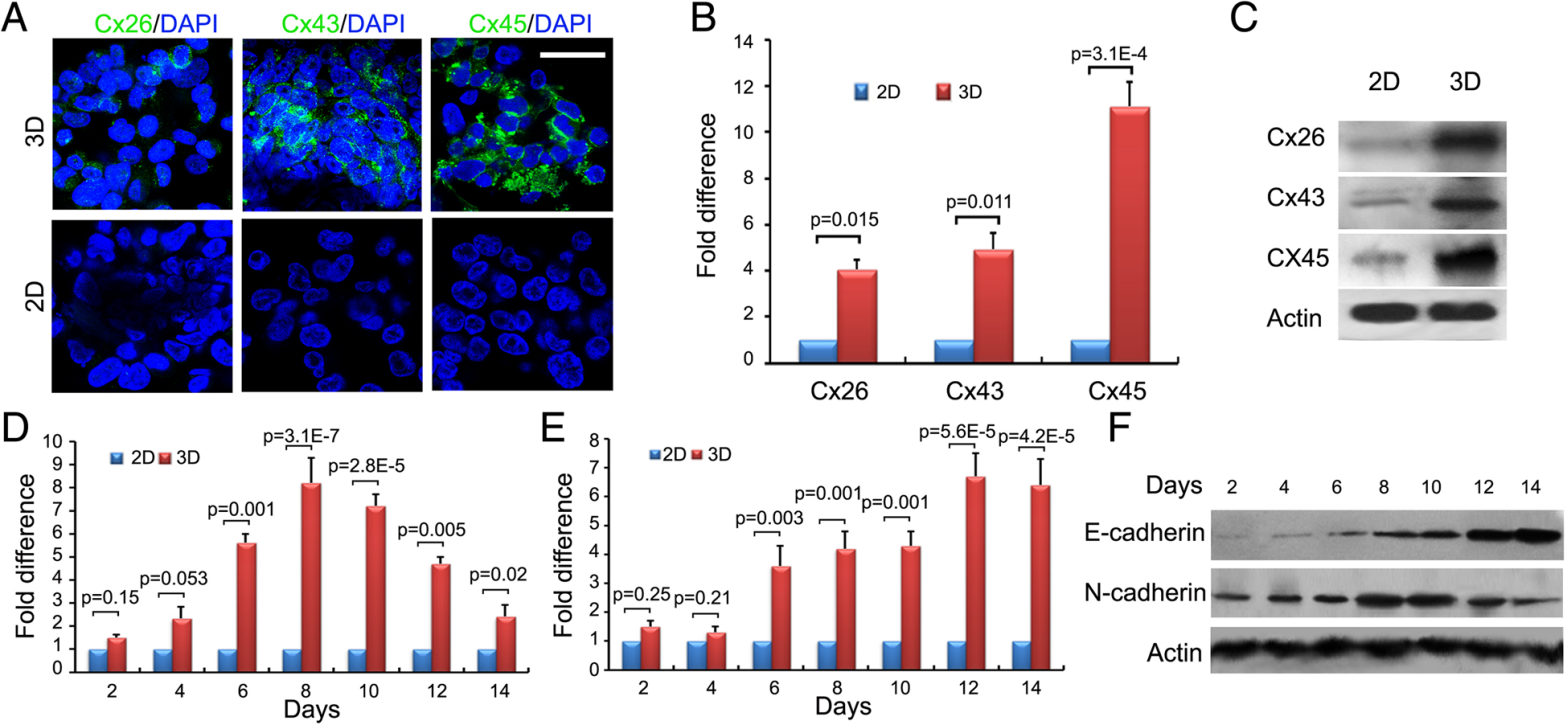
Differences in adhesion and gap junction molecule expression of HOSS1 cells in 3D and 2D cultures. **a**: Immunofluorescence staining of Cx26, Cx43, and Cx45 in 3D (upper) and 2D (lower) cultures of HOSS1 cells. Nuclei were counterstained with DAPI. Bar = 20 μm. Magnification, ×400. **b**, **c**: RT-qPCR and western blot analyses revealed up-regulation of the mRNA and protein expression of connexins in 3D cultures, respectively. **d**, **e**: mRNA expression of N-cadherin (**d**) and E-cadherin (**e**) in HOSS1 cells as determined by RT-qPCR. **f**: Western blotting showed up-regulation of E-cadherin in a time-dependent manner in 3D cultures, whereas N-cadherin expression reached a peak day 10 and decreased thereafter. Results represent the mean ± SD of three independent experiments with Student’s *t*-test

To assess cellular interactions, we detected the expression of adhesion and gap junction molecules in cells after 8 days of spheroid formation. First, gap junctions were identified between adjacent cells. An abundance of Cx26, Cx43, and Cx45 was detected in HOSS1 cell spheroids, which were absent in 2D cultures (Fig. 2a). These results were supported by RT-qPCR and western blot analyses of HOSS1 cells (Fig. 2b, c). Interestingly, compared with 2D cultures, mRNA expression of N-cadherin in HOSS1 cells increased in 3D cultures during the initial phase of spheroid formation and then decreased after 8 days (Fig. 2d, e). In contrast, both the mRNA and protein expression of E-cadherin was weak in the earlier period of spheroid formation, and then increased at later stages when the cells had proliferated as spheroids after day 6 (Fig. 2f). In addition, because of the cell–cell contacts over the entire cell surface, E-cadherin protein expression was consistently higher in 3D cultures compared with 2D cultures in which cells only make limited contacts. All target genes were validated in other sarcoma cell, in which Cx26, Cx43, Cx45, E-cadherin and N-cadherin were enhanced both mRNA and protein levels (Additional file 4: Figure S2).

### Increases in ECM components of 3D cultures

**Fig. 3:**
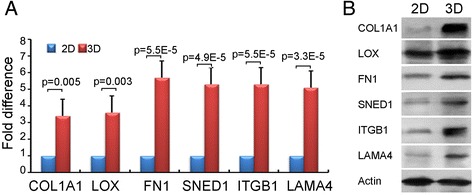
ECM-related gene expressions of HOSS1 cells in 3D and 2D cultures. **a**: RT-qPCR analysis of the expression of ECM-related genes in 3D and 2D cultures of HOSS1 cells at day 8. Data are presented as fold differences relative to 2D-cultured cells for each gene, which was defined as 1 (calibrator). Data are mean ± SD of 3 independent experiments with Student’s *t*-test. Error bars indicate SD. **b**: Western blotting of ECM-related protein expression in 3D and 2D cultures at day 8

Next, we detected the gene and protein expression of stromal ECM components such as collagen and fibronectin-1. mRNA expression of *COL1A1*, *LOX*, *FN1*, *SNED1*, *ITGB1*, and *LAMA4* was examined by RT-PCR at various time points in 3D and 2D cultures (Fig. 3a). The expression of these genes was higher in 3D cultures during sphere formation and the compaction phase. In confluent 2D cultures, the expression of these genes did not increase. Expression of collagen I and fibronectin-1, which mediate cell and tissue cohesion, was confirmed by western blot analysis of spheroids. Lysyl oxidase (LOX), a proteoglycan with high expression in many cancerous tissues, is found in the intercellular and extracellular stroma of soft sarcomas. We detected high expression of LOX in 3D cultures, whereas LOX was only expressed in 2D cultures when the cells became confluent. Expression of SNED1 (sushi-nidogen and EGF-like domains 1) was strongly up-regulated in 3D culture (Fig. 3b). Interestingly, some studies have considered SNED1 as a cisplatin resistance-related gene in head and neck squamous carcinoma [19]. Therefore, it may possibly reflect chemo-resistance in 3D spheroids.

### Chemo- and radio-resistances in 3D cultures

**Fig. 4:**
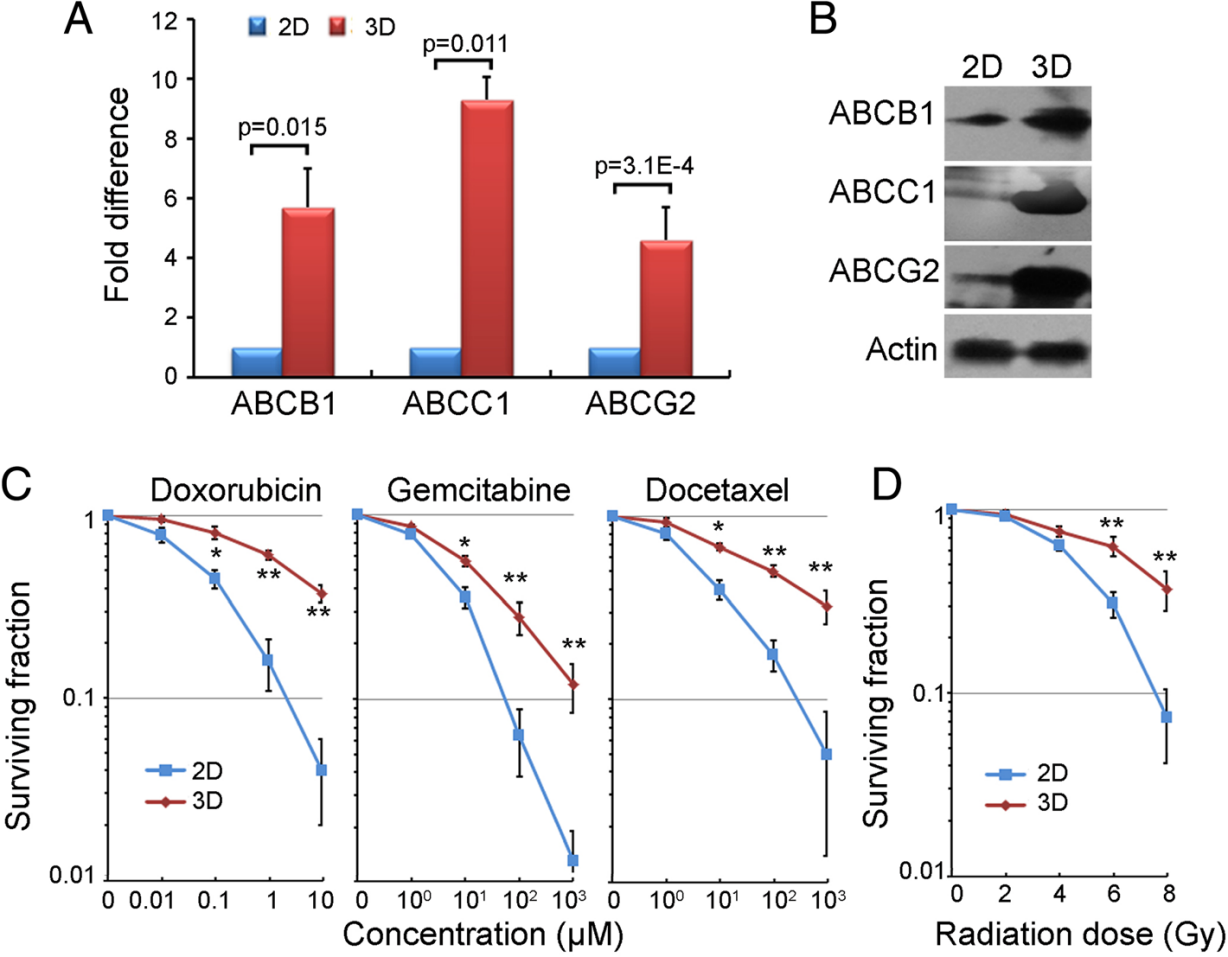
Increases in chemo-resistances against anticancer agents and radio-resistances in 3D cultures of HOSS1 cells. **a**: RT-qPCR analysis of drug resistance-related genes (ATP-binding cassette transporters) in 3D and 2D cultures. Values in 2D cultures for each gene were defined as 1. Results are the mean ± SD (*n* = 3) with Student’s-*t* test. **b**: Western blot analysis of HOSS1 cells in 3D and 2D cultures. **c**, **d**: Clonogenic survival of 3D- and 2D-cultured cells exposed to various concentrations of Doxorubicin (10, 100 nM, 1 μM, and 10 μM), Gemcitabine (1, 10, 100, and 1000 μM), and Docetaxel (1, 10, 100, and 1000 μM) (**c**) or X-ray doses (2, 4, 6, and 8 Gy) (**d**) at 24 hours after plating. ^*^ *P* < 0.05; ^**^ *P* < 0.01 vs. respective 2D-cultured cells. Results of a representative independent experiment of three are shown

We detected the mRNA and protein expression of ABCB1, ABCC1, and ABCG2 in 3D cultures for comparison with 2D cultures. Most of these ABC transporters were up-regulated during spheroid formation (Fig. 4a, b). Next, we examined the differences in the sensitivities to Doxorubicin [20], Gemcitabine [21] and Docetaxel [22] applied to 3D and 2D cultures. Each clone formation of the three drugs was detected in both 2D and 3D cultures of HOSS1 cells (Fig. 4c). And IC_50_ of three drugs were calculated in 2D and 3D culture with all four soft sarcoma cell lines (see Table 1). The reasons are related to the specific interactions between cells and their microenvironments, namely cell-cell and cell-ECM interactions, which maintain the effects of chemotherapy. Additionally, we clarified the effect on radio-resistance under 3D conditions. Cells in 3D culture had significantly higher clonogenic survival upon exposure to increasing single doses of X-ray radiation from 1 to 8 Gy compared with 2D cultures (Fig. 4d, Table 2).

**Table 1:** Comparative analysis of IC_50_ (μM) values of various anticancer drugs in 2D and 3D cultures

Cells	Doxorubicin		Gemcitabine		Docetaxel	
	2D	3D	2D	3D	2D	3D
HT1080	0.025 ± 0.002	4.27 ± 0.38*	4.26 ± 0.58	39.26 ± 2.76*	2.88 ± 0.54	69.81 ± 5.50*
RD	0.014 ± 0.01	2.74 ± 0.56*	1.89 ± 0.64	43.27 ± 3.32*	1.17 ± 0.16	55.87 ± 6.61*
SW872	0.066 ± 0.04	2.45 ± 0.72*	2.57 ± 0.15	18.34 ± 2.26*	1.85 ± 0.22	94.48 ± 10.25*
HOSS1	0.078 ± 0.011	4.61 ± 0.33*	6.23 ± 0.19	23.55 ± 3.65*	6.72 ± 0.87	103.2 ± 7.82*

Each data point is represented as mean ± SD (*n* = 4-6)

*P < 0.01 Vs repective 2D cultures

**Table 2:** Radio-sensitivity parameters of different cell lines in 2D and 3D cultures

Cells	SF2		D		N	
	2D	3D	2D	3D	2D	3D
HT1080	0.78	0.95	1.51	2.27	2.7	4.8
RD	0.81	0.91	2.34	2.74	2.1	4.6
sw872	0.77	0.84	1.86	3.45	2.8	5.1
HOSS1	0.89	0.94	2.28	3.8	2.6	4.8

SF2: cell survival fraction in 2Gy irradiation dose; Do: mean lethal dose value; N: extrapolation number

### Apoptosis of 3D spheroids

**Fig. 5:**
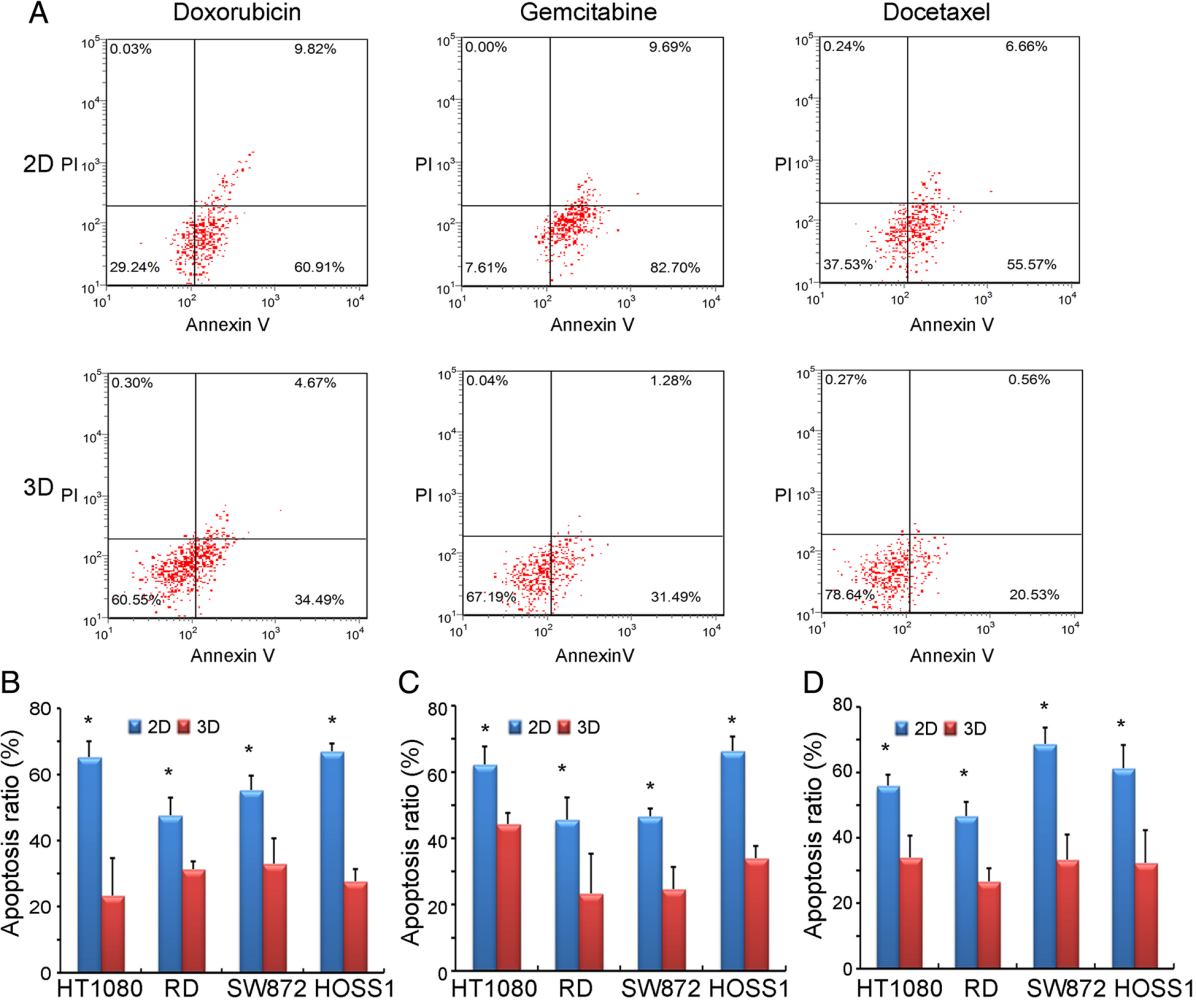
Apoptosis of soft sarcoma cells treated with anticancer agents in 3D and 2D cultures. **a**: Flow cytometric analysis of apoptotic HOSS1 cells in 2D (upper) and 3D (lower) cultures treated with 0.02 μM Doxorubicin, 2 μM Gemcitabine, or 2 μM Docetaxel (Annexin V positive: apoptotic cells; PI positive: dead cells.) About 2–3 fold fewer HOSS1 cells were apoptotic in 3D cultures than in 2D cultures. **b**, **c**, **d**: HT1080, RD, SW872, and HOSS1 cells were exposed to Doxorubicin (**b**), Gemcitabine (**c**), or Docetaxel (**d**) in 3D and 2D cultures. Apoptosis ratios were calculated from three independent experiments for all four cell lines. ^*^ *P* < 0.05 vs respective 2D-cultured cells

In 2D cultures treated with drugs, we observed a significant decrease in cell proliferation as well as an increase to 50–70 % apoptotic cells in the four sarcoma cell lines. To quantify apoptosis induced by Doxorubicin, Gemcitabine and Docetaxel, we used an Annexin V and PI analysis kit. Remarkably, induction of apoptosis was observed after 24 h with differences in the sensitivities of HOSS1 cells to the three drugs in 3D and 2D cultures (Fig. 5a). The apoptosis ratio was higher (55–80 %) in 2D cultures than in 3D cultures (20–35 %) at the same drug concentrations. Furthermore, similar sensitivities of the four sarcoma cell lines to Doxorubicin, Gemcitabine, and Docetaxel were found in 3D cultures (Fig. 5b, c, d). HT1080, RD, SW872 and HOSS1 cells showed significant decreases in apoptosis by about 20–45 % upon drug treatments at high doses in 3D culture. These data showed a limited response of apoptosis upon high-concentration drug treatments in most soft sarcoma cells in 3D culture.

## Discussion

Because most experiments have used cells grown in 2D monolayer cultures, in this study we analyzed the effect of cell–cell communication and more physiological settings on soft sarcoma formation by employing a 3D culture system. In 3D platform, sarcoma cell growth more closely mimicked the in vivo pathological process [23]. The cells appeared to be healthy when cultured in the 3D environment in vitro under our cultured conditions. We employed culture supplements and methylcellulose, but not matrigel or agarose, because these factors did not facilitate trypsinization of spheroids to count the number of cells accurately. Certain growth factors used in stem cell culture medium, such as EGF and bFGF, were used to prevent cell differentiation and maintain cell growth [24]. In this system, cell spheres formed well, but the time needed for cell growth stableness (in logarithmic growth phase) was about 8 days in 3D culture, whereas only 2 days were required in 2D culture, because the single cells needed more time to adapt to the 3D environment. Upon re-establishment of cell–cell communication in spheres, the cell signals are distributed appropriately, and the cells grow in more similar manner as their original source.

Using gene chip analysis, we identified 116 genes that were up-regulated and 94 genes that were down-regulated in 3D culture compared with 2D cultured cells. These genes belong to the various categories of signal transduction, transcription factor, cell cycle, cell growth, motility, apoptosis and differentiation, as well as gap junction, cell adhesion and ECM compositions.

Beginning, in our 3D model, we found expression of connexins Cx26, Cx43 and Cx45, which affected the proliferation of sarcoma cells, whereas their expression was lost in 2D culture. Many studies have shown that gap junction molecules such as Cx43 contribute to cell–cell interactions, although their expression differs in various cell types [25–27]. Currently, it is unclear why connexins were re-expressed in 3D culture. However, immunofluorescences staining showed expression of gap junction proteins in 3D culture were raised up, indicating a change in the cellular phenotype. It suggests that the expression of connexins depends on the environment and the effects of biological factors.

Besides, the expression of cadherin, cell–cell adhesion molecules, has been reported in some sarcomas. Both E-cadherin and N-cadherin are weakly expressed in sarcoma in 2D cultures as reported previously [28–30]. However, in our 3D cultures, both of these molecules were up-regulated and increased the cell–cell communication of sarcoma cells. There were also cell line-specific differences that further highlight the importance of testing the effects of various factors in a proper 3D environment.

Last, when cells form spheres, the composition of the ECM, such as collagen, laminin, and fibronectin, contribute to the remolding of ECM. For example, it has been reported that laminin A4 is associated with metastasis in some soft sarcoma cells according to influence tumor microenvironments [31]. Biologically active molecules related to the composition of the microenvironment, such as LOX, have been shown to be up-regulated in soft sarcoma cells [32, 33]. It would be functionally cross-linking collagen to contribute increasing the stiffness in 3D culture. It is the reason that the remolding of ECM is so necessary to 3D cultures of sarcoma cells.

On functional level, the growth response of cells to chemotherapeutic drugs and radiation treatment in 3D culture was similar to that of cells in 2D culture. However, we observed more chemo and radio resistance in medium containing methylcellulose of 3D culture. These data could be explained by several factors. First, the restoration of defective gap junctions in cancer could promote re-sensitization to current chemotherapeutics [34]. In human spheroidal cultures, gap junctions were identified as mediators of tumorigenicity by influencing E-cadherin expression [35]. Second, loss of E-cadherin involved in the regulation of EMT and metastasis, and its roles in both drug and immune resistance point out that Snail is a target for sensitization to cytotoxic drugs in cadherin signal pathway [36]. Third, remolding of ECM is benefited for protecting cells from chemotherapy and radiotherapy [37–39]. These factors contribute to chemo- and radio-resistance depended on the microenvironments in 3D culture.

Furthermore, in addition to factors in the stromal microenvironment, ABC transporters play an important role in chemo-resistance by pumping out drug molecules [40]. Three major ABC transporters, ABCB1, ABCC1, and ABCG2, were upregulated at both gene and protein levels in 3D cultures, which contributes to chemo-resistance. In our study, the cellular response to drugs was affected by microenvironmental factors, and 3D culture appeared to be more appropriate to detect chemo-resistances. Our results confirmed increases in the chemo-resistance of the four sarcoma cell lines. The reason why higher concentrations of drugs, which are used to treat sarcoma in clinical therapy, were present in the 3D model is possibly explained by more abundant ABC transporters and the enhanced biologically active molecules in 3D culture. Acquiring new phenotypes as a result of alterations in the microenvironmental composition is a major factor protecting cells from drug activities. Moreover, we examined radio-resistance against X-rays. Most soft sarcomas are not sensitive to X-ray irradiation, but their resistance increases in growing spheroids [41]. Our data suggest a connection between growth conditions and cellular chemo- and radio-resistances.

Accumulation of Doxorubicin, Gemcitabine, and Docetaxel caused apoptosis as detected by Annexin V and PI staining. The three drugs could not sensitize cells to undergo the early period of apoptosis in 3D culture, but contributed to a significant increase in apoptosis of cells in 2D cultures. The dysfunction of apoptosis may result from growth in the 3D environment, which is probably the reason for the radio-resistance.

In summary, 3D culture models are being increasingly employed in cancer research, as well as in tissue engineering and developmental and cell biology. To develop therapies, cell responsiveness is an important issue, and there are major differences in 3D physiological microenvironments and 2D culture. Why does the positive anticancer effect observed in 2D cultures often fail during in vivo testing? One of the reasons is that we ignored cell-cell communications in tumor spheres formation. As the results, the ability to predict outcomes in preclinical animal experiments and clinical trials can be better understood using 3D cultures, which is a potentially useful predictive tool in clinic anti-sarcoma screening in the future. Using 3D spheroid cultures from clinic surgical patients can be expanded to various fields of drug sensitive test, radio sensitive test, novel drug delivery approaches and X-ray project design for personal anti-sarcoma therapeutic strategy.

## Conclusion

Presently, a limitation of the 3D model is the number of cell populations and supplements. Accordingly, this model requires more refined tools to examine the adhesion, gap junction, ECM and apoptosis associated with drug- and radio-resistances in soft sarcoma.

## Additional files

Additional file 1:
**Supplemental Methods.**
Additional file 2:
**Supplemental Tables. PCR primers, Antibodies and Gene Chip Data.**
Additional file 3: Figure S1. Primary culture HOSS1 cell line from soft-sarcoma PDX. (A): Representative photograph shown soft-sarcoma formation in NOD/SCID mice from patient derived xenograft.Arrow shown sarcoma formats by subcutaneous injection. (B): The histology of PDX tissues was verified from original patient by H&E staining. (C): morphology of HOSS1 cell line was taken by phase contrast microscope. Bar = 50 μm, Magnification, ×400. Additional file 4: Figure S2. mRNA and protein levels in sarcoma cells. (A), (B) and (C): mRNA levels of target genes were shown respectively in HT1080, RD and SW872. (D): The proteins were expressed of HT1080, RD and SW872. Results represent the mean ± SD of three independent experiments with Student’s *t*-test.

## Abbreviations

HOSS1: Human Osteosarcoma cell line-1
PDX: Patient Derived Xenograft
FACS: Fluorescence-activated cell sorting
PI: Propidium iodide
ECM: Extracellular matrix
BPE: Bovine pituitary extract
LOX: Lysyl Oxidase, IC_50_, The 50 % inhibitory concentration
