# Supplementary material for: Associations of chemo- and radio-resistant phenotypes with the gap junction, adhesion and extracellular matrix in a three-dimensional culture model of soft sarcoma
Source: J Exp Clin Cancer Res. 2015 Jun 10;34(1):58. doi: 10.1186/s13046-015-0175-0 (PMC4467058; doi:10.1186/s13046-015-0175-0)
Supplement: Additional file 1: — Supplemental Methods. [file 13046_2015_175_MOESM1_ESM.docx]

Supplemental Methods:

**Establish PDX model and Primary culture from PDX**

**Step1:** PDX model forming: Residual sarcoma was taken at the time of surgery from previously untreated patients undergoing definitive surgery. Upon arrival in the pathology suite, the tissues were transported immediately to the animal facility in sterile RPMI medium. Then the whole sarcoma was minced into about 2mm^3^ pieces. The original patients’ sarcoma (F0 generation) was implanted subcutaneously into the flanks of 6-week-old NOD-SCID mouse with equal volume cold matrigel (BD, US) used 17 needle. When the resulting sarcomas grew to 1 cm^3^, each sarcoma (F1 generation) was resected and divided as for the primary sarcoma and passage into 5 mice (F2 generation) [[1](#_ENREF_1)]. The process was repeated to produce subsequent generations. The sarcomas and the derived PDXs were named human osteosarcoma HOSS; all PDX models maintained the same HOSS number as the parent sarcoma from which they were derived. All remaining sarcomas including F0 generation were snap-frozen in liquid nitrogen or stored in RNAlater (Life Technologies, CA, US) in order to protein and RNA analysis.

All animal studies were performed in accordance with the policies of the Institutional Animal Care and Use Committee and were approved by the Institutional Review Board of Peking University School of Oncology (No. 2014 KT 06).

**Step2**: Human osteosarcoma specimens were surgical operated from PDX of NOD/SCID mice. Fresh tissues were mechanically minced by Medi-machine (BD, US) and collagenase IV (1mg/ml, Sigma-Aldrich, US) digested 2hr at 4°C. Primary culture in RPMI 1640 medium without fetal bovine serum, containing 20ng/ml epidermal growth factor, 20ng/ml basic fibroblast growth factor, 10ng/ml hepatocyte growth factor(Invitrogen，US) over night at 37°C with 5% CO2. One week later, FBS-free medium were changed by the complete medium concluding 10% FBS, 100U/ml penicillin, 100μg/ml streptomycin (Invitrogen, NY, US) in a humidified atmosphere of 5% CO2 at 37°C, and expansion after removal fibroblasts. All tissues were acquainted by patients and uses of these tissues were approved by the Ethics Committee of Peking University Cancer Hospital (No. 2013 CT 03).

**3D culture system condition**

For preparation of methylcellulose stock solution we autoclaved 6 grams of methylcellulose powder (M0512, Sigma-Aldrich, US) in a 500 ml flask containing a magnetic stirrer (the methylcellulose powder is resistant to this procedure). The autoclaved methylcellulose was dissolved in heated 250 ml PBS (121°C) for 20 min. Thereafter, 250 ml free-FCS medium (room temperature) was added to a final volume of 500 ml and the whole solution mixed overnight at 4°C. The final stock solution was aliquot and cleared by centrifugation (5000 g, 2 h, room temperature). Only the clear highly viscous supernatant was used for the spheroid assay (about 90-95% of the stock solution). For spheroid generation, the stock solution was fleshly mixed with growth factors and supplements before used.

**Reverse Transcriptase**

cDNA was prepared with M-MLV Reverse Transcriptase kit according to the instructions of the manufacturer (Invitrogen). Briefly, cDNA was synthesized in a 20µl volume containing 1µg of DNase-treated total RNA, 1µl of oligo (dt)15 (50 µM), and 1µl 10 mM dNTP Mix, 4µl of 5-fold First Strand buffer, 1µl of 0.1 M DTT, 1µl of RNase OUT, and 1µl M-MLV. RNA, dNTPs and oligo(dt) primer were mixed first, heated to 65℃ for 5 min, and placed on ice until addition of the remaining reaction components. Then, the reaction mixture was incubated at 55℃ for 45 minutes and terminated by heat-inactivation at 70℃ for 15 minutes.

**qPCR reacted condition and amplification**

Each PCR reaction mixture of 10µl contained 1 µl of template cDNA (diluted 1:4), 5μl of SYBR-Green reagent, 1µl each specific primer (final concentration 0.4 µM) and 3μl sterile water. Thermal cycle protocols were performed for 40 cycles of 10 s at 95°C, 30 s at 60°C. All PCR reactions were carried out in triplicates using 7500 FAST Real time System (ABI, US). To confirm amplification specificity, PCR products were subjected to a melting curve analysis.

Reference:

1. Mosakhani N, Guled M, Leen G, Calabuig-Farinas S, Niini T, Machado I, Savola S, Scotlandi K, Lopez-Guerrero JA, Llombart-Bosch A et al: An integrated analysis of miRNA and gene copy numbers in xenografts of Ewing's sarcoma. Journal of experimental & clinical cancer research : CR 2012, 31:24.
