# Supplementary material for: Associations of chemo- and radio-resistant phenotypes with the gap junction, adhesion and extracellular matrix in a three-dimensional culture model of soft sarcoma
Source: J Exp Clin Cancer Res. 2015 Jun 10;34(1):58. doi: 10.1186/s13046-015-0175-0 (PMC4467058; doi:10.1186/s13046-015-0175-0)
Supplement: Additional file 2: — Supplemental Tables. PCR primers, Antibodies and Gene Chip Data. [file 13046_2015_175_MOESM2_ESM.zip › supplemental Table1.docx]

Supplemental Table 1.

PCR primer sequences

| qRT-PCR primers | | |
| --- | --- | --- |
| Gene Name | Sense | Anti-sense |
| *Cx26* | *5’-GAGTGAATTTAAGGACATCG-3* | *5’-GAAGACGTACATGAAGGCG-3* |
| *Cx43* | *5’-TCAACTGCTGGAGGGAAGGT-3* | *5’-AGATGAGCAGTCTGCCTTT-3* |
| *Cx45* | *5’-TGTCTGTTATGATGCGTTTG-3* | *5’-TTGTCTGCTTCACCGTGCT-3* |
| *CDH1* | *5’-TCCATTTCTTGGTCTACGCCT -3’* | *5’-TCACCTTCAGCCATCCTGTTT -3’* |
| *CDH2* | *5’-GTGCCATTAGCCAAGGGAAT-3’* | *5’-ATACTCACCTTGTCCTTGCG-3’* |
| *COL6A1* | *5’-CCTGGAGGGCTACAAGGAA-3'* | *5’-GTGCTTGGCCTCGTTCAC-3'* |
| *COL1A1* | *5’-CACACGTCTCGGTCATGGTA-3'* | *5’-AAGAGGAAGGCCAAGTCGAG-3'* |
| *ABCB1* | *5’-TGTCAAGGAAGCCAATGCCT-3'* | *5’-TCTGCCCACCACTCAACTGG-3'* |
| *ABCC1* | *5’-TGGGCAGGGATTCTCTTTTA-3'* | *5’-TCATGCTCACTTTCTGGCTG-3'* |
| *ABCG2* | *5'-GGAGGCCTTGGGATACTTTGAA-3'* | *5'-GAGCTATAGAGGCCTGGGGATTAC-3'* |
| *ABCC5* | *5’-CTGCTGTTTCCAAGGCATCT-3'* | *5’-GTGAGGGAGAGAACCAGCAC-3'* |
| *LAMA4* | *5’-TGGATGAGGAGGCAGATGA-3'* | *5’-GAGTGCGGGTCTCATTGTGC-3'* |
| *SNED1* | *5’-GCTCGAGAACATGGAGGAAG-3'* | *5’-CAGTTGCCAGGGACGTTT-3'* |
| *FN1* | *5’-GGTGGAATAGAGCTCCCAGG-3'* | *5’-GCAGCCTGCATCTGAGTACA-3'* |
| *LOX* | *5’-GTTCCAAGCTGGCTACTC-3'* | *5’-GGGTTGTCGTCAGAGTAC-3'* |
| *ITGB1* | *5’-CCATTGACCTCTACTACCT-3'* | *5’-CCTCATTTCATTCATCAG-3'* |
| *GAPDH* | *5'-GACCCCTTCATTGACCTCAAC-3'* | *5'-CTTCTCCATGGTGGTGAAGA-3'* |
