# Supplementary material for: Associations of chemo- and radio-resistant phenotypes with the gap junction, adhesion and extracellular matrix in a three-dimensional culture model of soft sarcoma
Source: J Exp Clin Cancer Res. 2015 Jun 10;34(1):58. doi: 10.1186/s13046-015-0175-0 (PMC4467058; doi:10.1186/s13046-015-0175-0)
Supplement: Additional file 2: — Supplemental Tables. PCR primers, Antibodies and Gene Chip Data. [file 13046_2015_175_MOESM2_ESM.zip › Supplemental Table2.docx]

Supplemental Table2

Information for antibodies used in Western blots

| Name | Vender | Cat No. | Species | Dilution |
| --- | --- | --- | --- | --- |
| Connexin26 | Abcam | ab65969 | Rabbit polyclonal IgG | 1:1000 |
| Connexin43 | Abcam | ab117843 | Rabbit polyclonal IgG | 1:2000 |
| Connexin45 | Santa Cruz | sc25716 | Rabbit polyclonal IgG | 1:2000 |
| E-cadherin | Santa Cruz | sc52328 | Mouse monoclonal IgG | 1:2000 |
| N-cadherin | Epitomics | 1791-4 | Rabbit monoclonal IgG | 1:2000 |
| Fibronectin | Abcam | ab2413 | Rabbit polyclonal IgG | 1:2000 |
| Collagen I | Epitomics | 7785 | Rabbit monoclonal IgG | 1:2000 |
| SNED1 | Abcam | ab174157 | Rabbit polyclonal IgG | 1:1000 |
| LOX | Abcam | 94991 | Rabbit polyclonal IgG | 1:2000 |
| ABCB1 | Cell Signaling | 13978 | Rabbit monoclonal IgG | 1:2000 |
| ABCC1 | Santa Cruz | sc365990 | Mouse monoclonal IgG | 1:2000 |
| ABCG2 | Epitomics | 2099-2 | Rabbit monoclonal IgG | 1:2000 |
| Actin | Roche | 1378996 | Mouse monoclonal IgG | 1:10000 |
