# Supplementary figures and images for: Associations of chemo- and radio-resistant phenotypes with the gap junction, adhesion and extracellular matrix in a three-dimensional culture model of soft sarcoma
Source: J Exp Clin Cancer Res. 2015 Jun 10;34(1):58. doi: 10.1186/s13046-015-0175-0 (PMC4467058; doi:10.1186/s13046-015-0175-0)

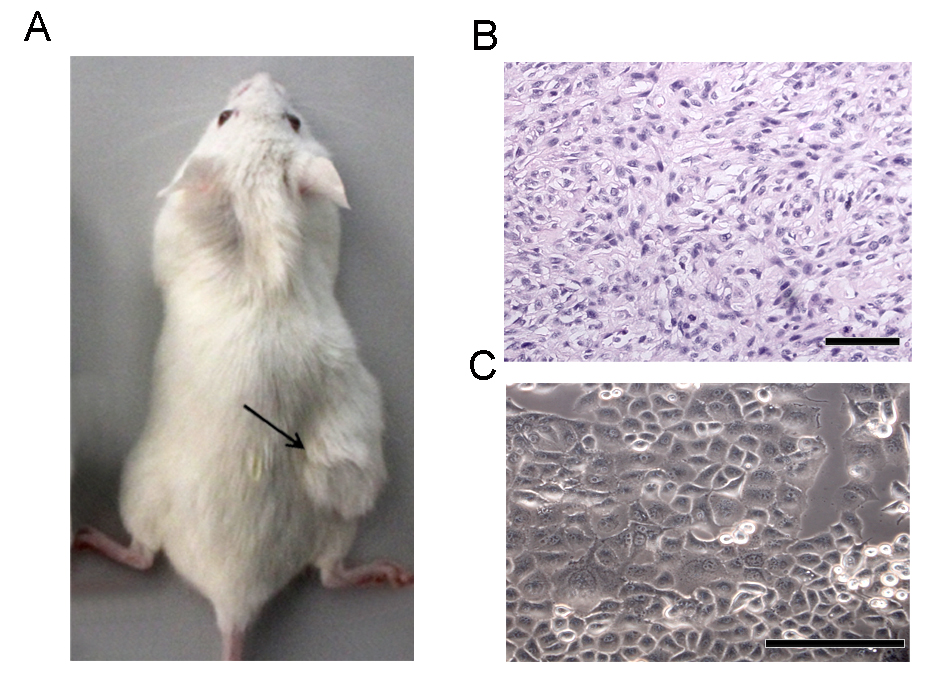

Supplement: Additional file 3: Figure S1. — Primary culture HOSS1 cell line from soft-sarcoma PDX. (A): Representative photograph shown soft-sarcoma formation in NOD/SCID mice from patient derived xenograft.Arrow shown sarcoma formats by subcutaneous injection. (B): The histology of PDX tissues was verified from original patient by H&E staining. (C): morphology of HOSS1 cell line was taken by phase contrast microscope. Bar = 50 μm, Magnification, ×400. [file 13046_2015_175_MOESM3_ESM.jpg]

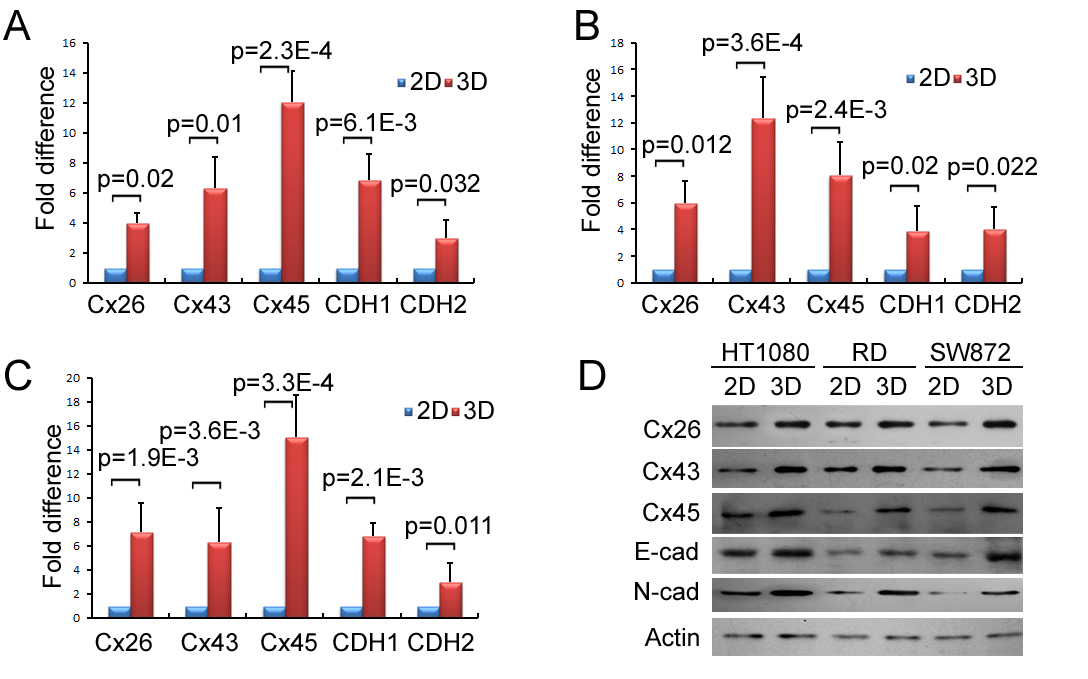

Supplement: Additional file 4: Figure S2. — mRNA and protein levels in sarcoma cells. (A), (B) and (C): mRNA levels of target genes were shown respectively in HT1080, RD and SW872. (D): The proteins were expressed of HT1080, RD and SW872. Results represent the mean ± SD of three independent experiments with Student’s t-test. [file 13046_2015_175_MOESM4_ESM.jpg]
